# Sustained Relief of Complex Regional Pain Syndrome (CRPS) Pain Following a 60-Day Peripheral Nerve Stimulation: A Report of Three Cases

**DOI:** 10.7759/cureus.54458

**Published:** 2024-02-19

**Authors:** Genaro J Gutierrez, Claire A Zurn, Nathan D Crosby

**Affiliations:** 1 Pain Management, Pain Specialists of America, Austin, USA; 2 Research and Development, SPR Therapeutics, Cleveland, USA

**Keywords:** chronic pain, 60-day pns, neurostimulation, neuromodulation, complex regional pain syndrome, peripheral nerve stimulation, crps

## Abstract

Patients who present to pain clinics with complex regional pain syndrome (CRPS) typically have debilitating pain, including hyperalgesia and allodynia, and additional substantial quality-of-life concerns related to the motor and autonomic-related symptoms of CRPS. Present treatments for CRPS such as neuropathic pain medications and sympathetic blocks are often unsatisfactory for managing symptoms. The present cases highlight the use of a 60-day percutaneous peripheral nerve stimulation (PNS) treatment for three patients with CRPS Type I affecting the foot. In all three patients, the tibial and common peroneal nerves were targeted separately at the popliteal fossa with two percutaneous leads each placed a remote distance (~1 cm) from the target nerve under ultrasound guidance. All three patients reported substantial pain relief and resolution of autonomic symptoms (e.g., swelling, edema, erythema), with sustained relief lasting 8-10 months in two patients, and 34 months (as of this writing) in the third patient. There were no medical complications. These three cases suggest that 60-day PNS is a safe and efficacious treatment for CRPS.

## Introduction

Complex regional pain syndrome (CRPS) is a chronic pain condition that can occur after an injury or tissue damage and is characterized by autonomic and functional changes in addition to enduring pain. The two subtypes of CRPS, Type I and Type II, are distinguished by the lack or presence of an identifiable nerve lesion, respectively [1]. Typically beginning in the distal portions of limbs, CRPS commonly includes classic hallmarks of neuropathic pain such as allodynia and hyperalgesia. CRPS also typically features autonomic and motor/trophic dysfunction including edema, sweating, temperature and skin color changes, decreased range of motion, and decreased function in the affected limb [2]. In the last two decades, the Budapest criteria have become the standard for the diagnosis of CRPS and based on these criteria, CRPS, including both subtypes, has an estimated annual incidence of 13.6-29.0 per 100,000 persons [1,3,4].

Conventional treatments for CRPS have typically included a combination of physical or occupational therapy, psychological therapy, neuropathic pain medications, and/or sympathetic nerve blocks [2]. The first line of treatments (physical, occupational, and psychological therapy) can help manage pain in CRPS patients, though for those with moderate to severe symptoms, these treatments often fall short [2]. Neuropathic pain medications have additionally been shown to improve pain and sleep in some patients, while interventions like sympathetic nerve blocks or sympathectomy lack strong evidence [2]. Permanently implanted neurostimulation therapies such as spinal cord stimulation (SCS), dorsal root ganglion stimulation (DRGS), and peripheral nerve stimulation (PNS) have advanced the treatment of CRPS in recent decades, particularly for those patients whose pain is refractory to more conservative approaches [2].

PNS in particular has been investigated for the treatment of CRPS with case reports dating back to the 1980s and 90s using conventional permanently implanted PNS approaches and often adapting SCS systems to use in the periphery [5]. More recently, a retrospective review of 165 patients receiving permanently implanted PNS over a thirty-year period showed decreases in pain and opioid use as well as improved function at a 12-month follow-up [6]. In most of these reported cases, PNS was trialed for two to ten days, and if a patient showed pain relief during that time, a permanent system was implanted. However, in addition to significant rates of complication such as infection or implantable pulse generator site pain, a permanently implanted system may not suit every CRPS patient’s needs [5,6]. For example, a fully implanted PNS system may be undesirable in younger patients or highly active patients. Further, a short trial prior to permanent implantation may be inadequate for some patients who may have presented as delayed responders or delayed non-responders to stimulation treatment given a longer, temporary stimulation period [7].

A 60-day PNS system has been developed in recent years to deliver treatment to a target nerve or nerves innervating the region of pain without the need for a permanent implant. The system includes leads placed percutaneously and connected externally to a body-mounted pulse generator. The percutaneous leads are designed with a coiled structure that enables tissue ingrowth to reduce dislodgement and infection risk, enabling treatment periods of up to 60 days [8]. This 60-day PNS treatment has shown success in treating various types of acute and chronic pain in clinical trials and retrospective analyses of real-world data, including reports of sustained relief of pain well beyond the treatment period in a majority of patients across various pain conditions [9-12]. As there are no published studies to date on the use of 60-day PNS specifically in patients with CRPS, the present work reports three cases of successfully treated CRPS Type I that highlight the potential of 60-day percutaneous PNS to provide significant, sustained pain relief, which enabled improvements in motor and autonomic symptoms and/or reductions in pain medication. All three patients provided written consent to be included in this report.

## Case presentation

Case 1

A 54-year-old woman presented to the clinic four months after fracturing her left second toe. In the months following the injury, she attempted bracing and physical therapy, but reported the development of burning and shooting pain, allodynia, and swelling throughout the foot. An MRI of the foot showed no other abnormalities and no orthopedic explanation for the severe pain. On examination, she exhibited the Budapest Criteria for CRPS, including edema, color and temperature asymmetry, and reduced range of motion in the ankle and toes. The patient did not report any limited sensation in the foot. After failing to see meaningful and durable relief of pain over six months with gabapentin, nortriptyline, tramadol, and three separate lumbar sympathetic blocks, the patient was offered a 60-day PNS treatment. At the start of PNS treatment, the patient was taking both gabapentin and nortriptyline.

**Figure 1:**
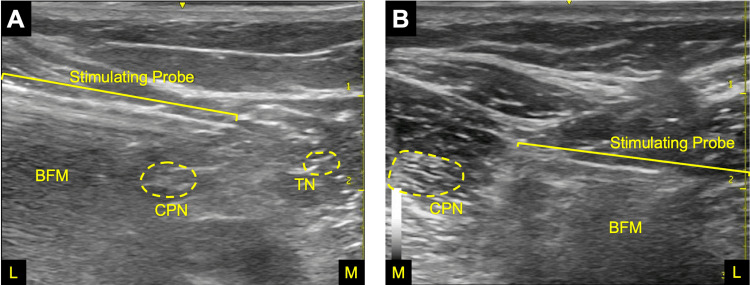
In-procedure ultrasound images of the stimulating test probe for targeting the common peroneal nerve (CPN) near the biceps femoris muscle (BFM) for Case 1 (A) and Case 2 (B). The tibial nerve (TN) was targeted separately with an additional lead for each patient. In both cases, the probe approached the nerve in a lateral (L) to medial (M) direction.

With the patient positioned prone, two percutaneous leads were implanted under ultrasound guidance near the tibial and common peroneal nerves at the popliteal fossa. Both leads were placed posterior (superficial) to the nerves with the electrode centered to cover the distribution of each nerve (Figure 1A). An external pulse generator was connected to the leads and programmed to deliver stimulation at 100 Hz. During and immediately after the procedure, the patient reported 100% coverage of the painful foot with comfortable stimulation-evoked sensations. Throughout the 60-day treatment, the patient controlled the intensity of stimulation with the latitude to deliver current amplitudes up to 30 mA and pulse widths from 10-200 μs. The patient was instructed to use the stimulation as much as possible up to 24 hours per day for the planned 60-day treatment and to adjust stimulation intensity as needed to maintain comfortable simulation-evoked sensations. There were no medical complications during the procedure, over the course of treatment, or when the leads were removed after two months from the initiation of treatment. During treatment, the patient ceased taking gabapentin and nortriptyline.

At the end of treatment, the patient reported 80% pain relief, and a reversal of edema and sudomotor changes in the foot were noted on the physical exam. The patient reported that she was once again able to wear shoes comfortably. The relief of symptoms was sustained at a follow-up visit one month after lead removal and the patient did not resume the use of gabapentin or nortriptyline. At ten months after the start of the PNS treatment, pain symptoms partially returned, though the patient remained significantly improved from baseline and pain was not significant enough to invoke interest in implantation of a conventional fully implantable PNS system.

Case 2

A 40-year-old female reported to the clinic with concerns of allodynia over her entire right foot, accompanied by color changes and swelling. No negative sensory signs were noted upon exam. She had difficulty sleeping due to pain induced when the bedsheets touched her foot. Three months prior, she fractured her third toe when a chair fell on her foot. She reported wearing a protective boot and attempting physical therapy during those three months, with neither resulting in a return to her state pre-injury. Upon physical exam, she met the Budapest Criteria for the diagnosis of CRPS. The patient responded very well to a lumbar sympathetic nerve block but had a return of pain after two months and had only modest pain relief with pregabalin, which she eventually ceased. Six months after the initial visit, the patient was offered a 60-day PNS treatment.

She received two percutaneous leads implanted to stimulate the tibial and common peroneal nerves using the same approach as Case 1 (Figure 1B), which she reported produced 100% coverage over her entire foot with comfortable sensations. Similar to Case 1, stimulation was delivered at 100 Hz at comfortable intensities for up to 24 hours per day. After two months of treatment, leads were removed from the body. There were no medical complications during lead placement, stimulation treatment, or lead removal. A lead fragment, which is magnetic resonance (MR) conditional, was retained in the body upon lead removal, with no sequelae to date.

Three months after lead removal, the patient continued to report 100% pain relief and the ability to sleep comfortably without pain. On physical exam, the swelling and erythema of the foot were resolved. As of this writing, 34 months after the start of PNS treatment, the patient continues to report pain relief and was able to resume teaching and coaching high school track and field.

Case 3

A 58-year-old female with a history of left foot and ankle operations began to report new pain in her left foot and lateral ankle. On examination, she met the Budapest Criteria for CRPS, including allodynia, edema, and reduced range of motion in the ankle and toes. She did not report any limited sensation in the foot. The pain failed to resolve with neuropathic pain medications, high-dose opioids, physical therapy, and nerve blocks. After these failed treatments and approximately four years after the foot and ankle surgeries, the patient received a fully implanted dorsal root ganglion (DRG) stimulation system. The DRG stimulation produced approximately 50% pain relief for the patient, and she reduced her daily opioid dose from 120 MME to 40 MME. After an additional four years, a 60-day PNS treatment was suggested.

Two leads were implanted percutaneously and targeted the tibial and common peroneal nerves. The patient reported 100% coverage of her region of pain with comfortable sensations. As per the instructions for use, the PNS system was placed such that the electrical current pathway did not overlap with that of the DRG stimulation system to avoid potential interference, and the DRG stimulation continued to be active during PNS treatment. After one month of stimulation, the patient reported an additional 75% pain relief from the level of pain when utilizing DRG stimulation alone. At the end of the two-month treatment, she reported completely resolved pain (100% relief overall) and resulting improved ability to stand, walk, and wear shoes. There were no medical complications in using the device.

Eight months after the start of PNS treatment, she continued to report 60% pain relief and had further reduced her daily opioid dose to 30 MME (from 40 MME at the start of PNS treatment). At 10 months post-treatment, the pain and opioid dose returned to baseline and the patient later elected to undergo a permanent PNS system implant with good results.

## Discussion

The cases present three adult patients successfully treated for CRPS in the foot with a 60-day percutaneous peripheral nerve stimulation treatment targeting the tibial and common peroneal nerves proximal to the location of the initial injury. The clinically significant and sustained reductions in pain also enabled improvements in motor and autonomic symptoms of CRPS, with the three patients further reporting various elements of improvement including being able to wear shoes, sleep comfortably, return to activities of daily living, or reducing pain medication dosage following the PNS treatment.

The complex combination of sensory, motor, and autonomic symptoms associated with CRPS has made it challenging to define the syndrome mechanistically, though researchers have identified several key characteristics. As with other nociplastic and neuropathic pain syndromes, peripheral and central sensitizations develop with hallmark symptoms including allodynia and hyperalgesia that persist past the time of healing of an initial injury or surgery [13]. Peripherally, a decrease in A⍺ and Aβ nerve fiber (non-nociceptive) signaling and/or morphological shift of these fiber populations to gain nociceptive functionality may diminish non-nociceptive input to the CNS. Additionally, sensitization of Aδ and C fiber pathways can result in elevated nociceptive signaling. Centrally, these somatosensory imbalances may produce cortical reorganization, with the representation of the affected limb shifting or diminishing relative to the unaffected limb [14]. Beyond the cortex, the changes in input may also result in decreases in volume and connectivity of deep brain structures, such as the putamen, which are thought to contribute to motor dysfunction [15]. Thus, the initial nerve injury may lead to anatomical changes in the central nervous system, resulting in CRPS symptoms such as allodynia, hyperalgesia, and motor dysfunction, including weakness and decreased range of motion.

In parallel with sensory and motor symptoms (e.g., dysfunction, weakness, and diminished range of motion), the autonomic symptoms of CRPS are also believed to evolve through both peripheral and central mechanisms. Autonomic and immune dysfunction may result from direct Aδ and C fiber links to the autonomic nervous system [2]. Beyond this physical coupling, mechanisms such as altered catecholamine circulation and increased expression and activation of ⍺1-adrenergic receptors in the affected limb may be responsible for the sudomotor and autonomic symptoms of CRPS [16]. Within the brain, changes in gray matter density and white matter connectivity suggest a central mechanism involved in autonomic, cognitive, and emotional symptoms in patients [17].

Studies suggest that the cortical reorganization associated with CRPS may be reversible or reconditioned in association with successful analgesic treatment. Maihofner et al. found that the resolution of CRPS symptoms was correlated with the reversal of the maladaptive reorganization in the somatosensory cortex [18]. Electrical stimulation may help drive beneficial cortical plasticity, and the 60-day PNS treatment used in the present cases has been proposed to recondition maladaptive cortical plasticity to produce long-term sustained relief. The system incorporates a large, monopolar lead that is proposed to robustly and selectively activate large diameter, non-nociceptive afferent fibers in the target nerve, proximal to the region of pain [19]. These robust increases in physiological non-nociceptive input, coupled with attenuation of nociceptive input (e.g., via the gating mechanism), may rebalance peripheral inputs to help recondition the CNS, producing sustained pain relief that persists long after the end of the stimulation treatment period [19].

The sustained relief of symptoms in the present cases supports the existence of a mechanism of action that enables long-term resolution of central sensitization following 60-day PNS. All three patients reported substantial long-term relief, and one patient continues to report relief of pain and the resulting improvements in other CRPS symptoms after 34 months. These outcomes of sustained relief are consistent with the results of published analyses of 60-day PNS in other pain indications, including both clinical and real-world data [9-12]. Post-amputation pain, especially phantom limb pain, is thought to be mechanistically linked to cortical reorganization in a similar manner to CRPS [20]. Long-term results from one randomized controlled trial treating post-amputation pain patients with 60-day PNS showed that 67% (6/9) of patients had sustained ≥50% reduction in pain at 12 months [10]. Additionally, in a retrospective survey of real-world patients who received 60-day PNS across a variety of nerves, a majority of treatment responders reported continued substantial reductions in pain and/or improvement in quality of life across follow-up periods ranging from three to more than 24 months [9].

Historical studies of permanently implanted neurostimulation systems for the treatment of CRPS have shown the potential to produce substantial pain and autonomic symptom relief [5,6]. The present cases highlight the potential for patients to achieve significant and sustained relief after lead withdrawal. A PNS treatment may be desirable in patients for whom permanent hardware is not preferred, such as younger or highly active patients. The present cases also highlight the potential of a 60-day PNS treatment to provide sustained relief while also providing information on whether a permanently implanted PNS system could be a viable option long-term if the pain returns. For example, in Case 3, the patient experienced a return of pain and eventually opted for a transition to a permanently implanted PNS system. Compared to the conventional seven to 10 days of trial stimulation prior to a permanent implant, recent real-world data have highlighted the benefits of a longer, 60-day treatment to help identify delayed responders to PNS [7]. The potential for significant and sustained pain relief and the ability to inform additional treatment strategies infer a benefit to using a 60-day PNS treatment for CRPS patients earlier in the care continuum.

## Conclusions

CRPS can be difficult to treat, with the complex combination of sensory, motor, and autonomic symptoms often proving refractory to conventional treatment options. While neuromodulatory treatments have shown promise, many require a fully implantable device and are thus used as a last resort treatment after physical therapy, medications, and sympathetic blocks. As the present cases demonstrate, a 60-day PNS treatment has the potential to successfully improve symptoms and, in some cases, may obviate the need for a permanently implanted system, making it a reasonable pain management treatment for patients with CRPS who have experienced failed conservative therapies.
